# Women, the intellectually humble, and liberals write more persuasive political arguments

**DOI:** 10.1093/pnasnexus/pgad143

**Published:** 2023-04-25

**Authors:** Jeffrey Lees, Haley Todd, Maxwell Barranti

**Affiliations:** Andlinger Center for Energy and the Environment, Princeton University, 86 Olden St, Princeton, NJ 08540, USA; School of Public and International Affairs, Princeton University, 20 Prospect Ave, Princeton, NJ 08540, USA; John E. Walker Department of Economics, Clemson University, 320 Wilbur O. and Ann Powers Hall, Clemson, SC 29634, USA; John E. Walker Department of Economics, Clemson University, 320 Wilbur O. and Ann Powers Hall, Clemson, SC 29634, USA; Department of Educational Studies, University of South Carolina, 820 Main Street, Columbia, SC 29208, USA; Department of Psychology, York University, 4700 Keele St, Toronto, ON M3J 1P3, Canada

**Keywords:** political persuasion, gender, ideology, intellectual humility

## Abstract

If sincere attempts at political persuasion are central to the functioning of democracy, then what attributes of individuals make them more persuasive toward fellow citizens? To examine this, we asked 594 Democrats and Republicans to write politically persuasive arguments on any topic of their choice and then gave those arguments to a US representative sample of 3,131 to rate the persuasiveness, totaling 54,686 judgments. We consistently found that arguments written by women, liberals, the intellectually humble, and those low on party identification were rated as more persuasive. These patterns were robust to controls for the demographics and partisanship of judges and persuaders, the topics written about, argument length, and the emotional sentiments of the arguments. Women's superior persuasiveness was partially, but not fully, explained by the fact that their arguments were longer, of a higher grade level, and expressed less dominance than men's. Intergroup dynamics also affected persuasiveness, as arguments written for in-party members were more persuasive than the ones written for out-party members. These findings suggest that an individual's personal and psychological characteristics durably provide them with a persuasive advantage when they engage in sincere attempts at changing the hearts and minds of fellow citizens.

**Significance Statement**

Democratic life requires political discussion and debate amongst citizens. In this study, we examined what types of individuals are more successful when asked to make a sincere attempt to politically persuade others. Using a large, online experiment, we found that the political arguments which tended to be more persuasive were arguments written by women, by people who acknowledge their own beliefs might be wrong, by people who identify as more liberal, and by people who identify as more politically independent. This was true whether the arguments were trying to persuade someone in one's own political party, or someone from the opposing party.

## Introduction

Political persuasion is core to a vibrant democracy where citizens engage in sincere attempts to sway the hearts and minds of others. Persuasion is a mechanism not only of attitude change over time (1), including reducing prejudices (2), but also of group polarization and radicalization (3). Understanding what persuader attributes predict persuasiveness across partisan lines, and within (4, 5), will advance knowledge of how interpersonal persuasion can help facilitate political tolerance (6).

Yet the durable persuasion effects of individual characteristics are difficult to isolate. In naturalistic settings, such as political advertisements (7), canvassing (2), and interpersonal exchange (8), attributes such as gender, race, and partisanship are highly endogenous with context, the persuader–receiver relationship, and receivers’ biases (e.g. race and gender biases; 9). Conversely, experimental settings can be too constrained to allow the pathways by which individual factors affect persuasiveness to emerge. For example, persuaders may be assigned to a single topic, or in- versus out-party effects may not be examined in tandem. Building on methodologies from social perception research (10), the present study was designed to observe individual characteristics that predict perceived persuasion for in- and out-party communication, while controlling for confounding factors. We find that identifying as female, being more liberal, being less identified with a party, and being more intellectually humble (the degree to which people recognize their beliefs might be wrong) are durably associated with perceived persuasiveness and that this cannot be completely explained by the topics written about, the language used, the identity or biases of judges, or other characteristics of the persuaders.

We asked an online sample of 597 Democrats and Republicans (persuaders) to write a persuasive argument on their choice of political topic. We asked, but did not require, persuaders to write at least four sentences. Persuaders were randomly assigned to one of three conditions: persuade an “average” in-party member, an “average” out-party member, or an “average American.” This manipulation allowed us to model how perceived persuasion varied by intergroup dynamics. Persuasion was financially incentivized, as base pay ($3.25 for 15 min) was doubled for those in the top 25% of perceived persuasiveness (within condition). The 597 arguments were given to a nonprobability US representative sample of 3,131 receivers (judges), each of whom rated six random arguments (appropriate to condition and party) on a three-item measure of perceived persuasiveness^1^ (11), totaling 54,686 judgments of perceived persuasiveness across 18,238 persuader–judge pairs. The only information judges had about persuaders was their party affiliation (which persuaders were aware of). Judges were unaware of the experimental condition or any other persuader attributes.

## Results

Perceived persuasion was modeled using linear mixed-effects regressions, with crossed random intercepts for persuader, judge, and the persuader–judge interaction (12), and fixed effects for all other variables. All analyses controlled for condition, persuaders’ age, gender, education, ethnicity, political identification, and judges’ political identification. No results presented here were contingent on the inclusion of control variables.

**Fig. 1.**
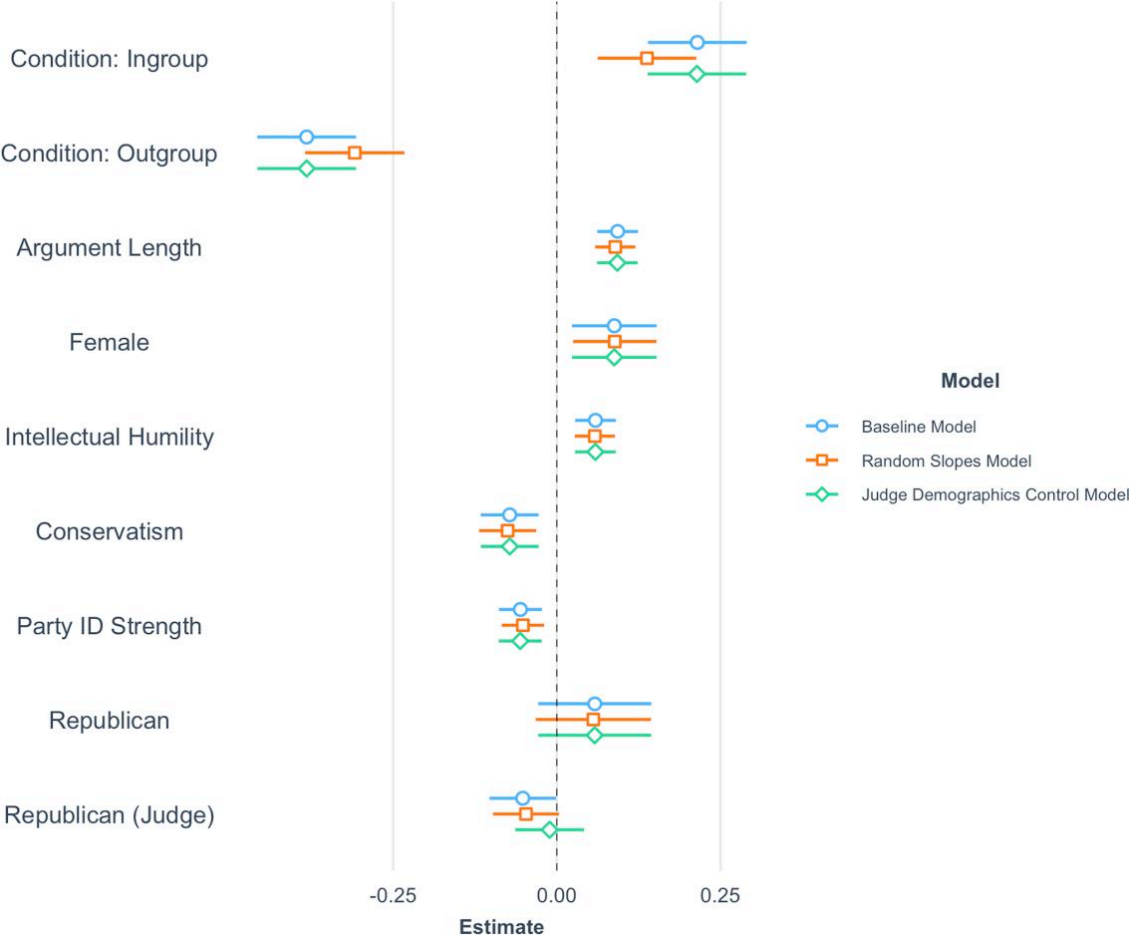
Standardized coefficients from mixture models predicting perceived persuasiveness, with 95% CIs. The baseline model includes random intercepts and controls for persuaders’ age, education, and ethnicity. The random slope models add random slopes within judge for condition and persuaders’ party identification. The judge demographic control model adds judges’ gender, age, ethnicity, and geographic region to the baseline model.

In the baseline model (see Fig. 1), condition strongly affected persuasiveness. Relative to the average American condition, arguments persuading out-party members were perceived as less persuasive, *β* = −0.38 (−0.46, −0.31), *t*(561.03) = −9.93, *P* < 0.001, and arguments persuading in-party members were perceived as more persuasive, *β* = 0.21 (0.14, 0.29), *t*(561.90) = 5.58, *P* < 0.001. Perceived persuasiveness was positively associated with intellectual humility, *β* = 0.06 (0.03, 0.09), *t*(565.14) = 3.72, *P* < 0.001, and argument length, *β* = 0.09 (0.06, 0.12), *t*(584.81) = 5.84, *P* < 0.001, and negatively associated with conservatism, *β* = −0.07 (−0.12, −0.03), *t*(567.11) = −3.19, *P* = 0.001, and absolute party identification strength, *β* = −0.06 (−0.09, −0.02), *t*(562.23) = −3.32, *P* < 0.001. Condition did not interact with the party identification of persuaders, *F*(2, 700.5) = 0.86, *P* = 0.425, or judges, *F*(2, 700.5) = 0.86, *P* = 0.425. All effects above were robust to controlling for judges’ demographics, and for modeling random slopes for condition and persuaders’ party identification within judge, to relax the assumption that judges’ responded to arguments from different conditions and partisans uniformly. There was also high rank-order agreement in the perceived persuasiveness of the arguments across all judges, two-way mixed ICC(C, *k*) = 0.83 (0.82, 0.84), *P* < 0.001.

**Fig. 2.**
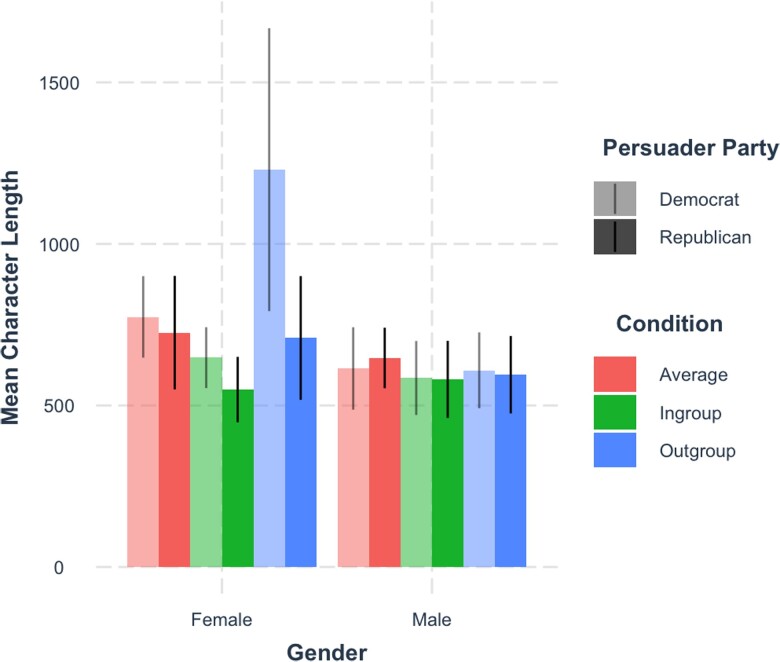
Barplot of mean argument character length by persuader's gender, party affiliation, and condition. Bars are 95% CIs.

Gender was consistently associated with perceived persuasiveness. In a model with condition, demographics, and party identification (judge and persuader), women's arguments were rated as more persuasive than men’s, *β* = 0.12 (0.05, 0.19), *t*(571.87) = 3.49, *P* < 0.001. This gender difference persisted in the baseline model, *β* = 0.09 (0.02, 0.15), *t*(565.28) = 2.66, *P* = 0.008, and therefore cannot be explained by the fact that women, compared with men, were more liberal, *β* = 0.21 (0.05, 0.37), *t*(591) = 2.58, *P* = 0.010, and wrote longer arguments (*M*_characters_women_ = 753, SD_char_women_ = 674, *M*_char_men_ = 608, SD_char_men_ = 423), *β* = 0.26 (0.10, 0.42), *t*(591) = 3.20, *P* = 0.001 (see Fig. 2), or other demographic factors. Gender did not interact with condition *F*(3, 564.1) = 1.91, *P* = 0.126, suggesting that women's arguments were rated more persuasive across all targets of persuasion.

We conducted exploratory sentiment analyses using *TextAnalyzer* (13), in an attempt to explain why women and liberals were higher in perceived persuasion. We first identified lexical attributes associated with perceived persuasion and gender (grade level and dominance) and persuasion and ideology (grade level, emotional valence, and fear). Adding the sentiment scores to the baseline model did not fully attenuate the gender and ideology effects. See Supplementary material for details.

**Fig. 3.**
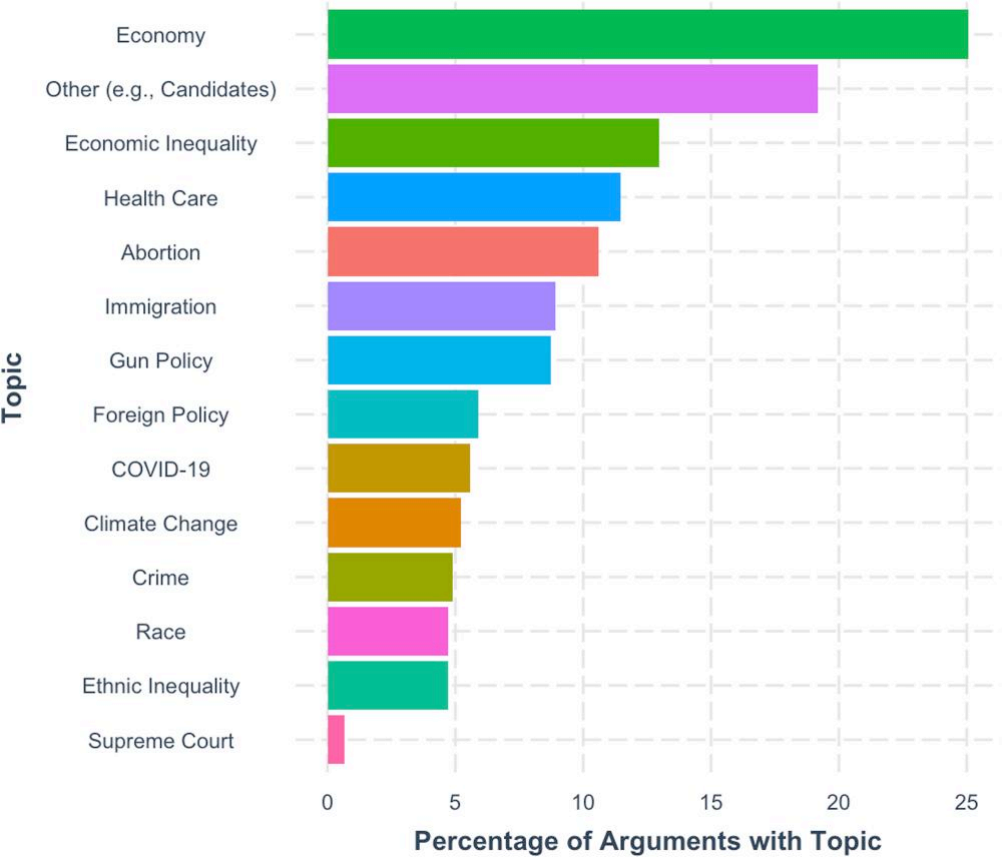
Plot of the nonexclusive percentage of arguments which contained each topic. Topics were coded by the research team. “Other” category included references to specific politicians, appeals to bipartisanship, and idiosyncratic issues.

**Fig. 4.**
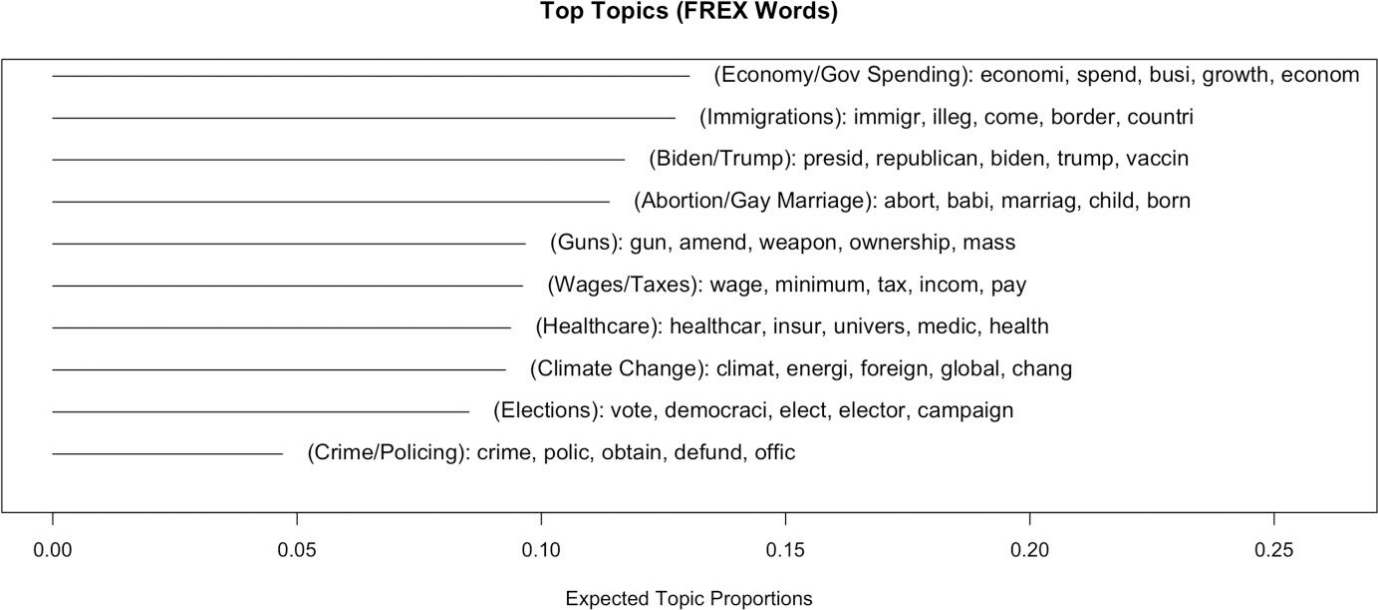
Summary plot of the expected frequency of each of the 10 topics across the corpus of arguments, based on a structured topic model. Labels in parentheses are researcher generated. Words displayed are FREX words, i.e. weighted by both frequency and exclusivity to the topic.

Lastly, we coded each argument for the presence of 14 political topics of concern to voters (see Fig. 3), taken from Pew Surveys (see Supplementary material). Controlling for argument topic in the baseline model, which explained significant variance, *F*(57, 507.9) = 1.61, *P* = 0.004, did not meaningfully affect any of the other relationships. Two categories (economic inequality and healthcare) were significantly higher in mean perceived persuasiveness than the average of all other categories, and one (other) was significantly lower (see Supplementary material). Gender was not independent of category choice, *χ*^2^(26, 764) = 109.69, *P* < 0.001, and women wrote more about economic inequality/healthcare and less in the other category than men did (see Table S1). As such, dummy variables for each of those three categories were added to the baseline model. None were significant, and the gender effect persisted (see supplementary material). Researcher-coded categories hued closely to the outputs of a structured topic model (14) (see Figs. 4 and S1).

## Discussion

We found that identifying as female, being liberal, having weak party identification, and being intellectually humble were all associated with perceived persuasiveness and robust to controlling for the demographic and political attributes of persuaders and judges, condition, and argument sentiments, topics, and length. Replicating past work, partisans were less amenable to arguments from the out-party relative to the in-party (15), though this effect could also be driven by inaccurate perceptions of what out-partisans actually believe (5). The inclusion of an intergroup target manipulation provides greater generalizability across real-world contexts of interpersonal persuasion, as persuasion can occur across and within party lines (4, 6). As judges were aware of persuaders’ party identification, we cannot rule out anticonservative prejudice driving the association between liberalism and perceived persuasiveness, despite controls for judges’ party identification. However, the lack of an interaction between condition and judges’ party identification suggests that this is not entirely driven by prejudice (i.e. not driven entirely by Democratic judges). Indeed, a core strength of the design was that judges were unaware of persuaders’ other characteristics, and therefore their associations with perceived persuasiveness *must* be due to judges’ reactions to the contents of the arguments. For example, the intellectual humility effect may be driven by persuaders who foreground the uncertainty of their beliefs, which can enhance persuasiveness (16). Our findings also align with work suggesting that persuasion effects using externally valid arguments will typically be small (17), as we find standardized estimates of *β* < 0.10 for most persuader characteristics.

Of notable interest was the persistent gender effect. Attempts to explain it motivated the sentiment analyses, yet despite gender differences in sentiment and argument length, these did not fully explain the gender effect. Perhaps our sentiment analysis did not capture characteristics which predict perceived persuasiveness and may vary by gender, for example using personal experience instead of facts when constructing an argument (18). Seminal work on interpersonal persuasion finds that persuader trait effects can covary with characteristics more directly tied to persuasion (e.g. physical attractiveness correlates with objective language fluency (19)), suggesting our gender effect may be driven by gendered socialization processes which affected persuaders’ choices about what to write and how. Future research should expound upon the roots of this gender effect.

Several limitations are of note. First, the persuaders sample was a convenience sample, which limits generalizability. Second, written persuasion may not generalize to spoken persuasion, such as canvassing (2), and the persuasiveness of laypeople's arguments on topics of their choosing may not generalize to elite political messaging (7). In interpersonal contexts, receivers likely know the gender of persuaders, while persuaders may not know the political affiliation of receivers. From an elaboration likelihood model perspective, our design privileged central over peripheral route processes relative to other forms of written persuasion, such as online communication (20), though our findings also suggest that factors that might be considered peripheral (e.g. gender) may actually be related to central route processes. Financially incentivizing participants to write persuasive arguments may also attenuate some intrinsic motivations for engaging in political arguments (21), which may limit generalizability. Lastly, we measured perceived persuasion—not actual attitude change, though the high rank-order agreement in perceived persuasiveness suggests the argument may track with actual attitude change (22). These limitations notwithstanding, we encourage researchers across disciplines to build upon these findings and uncover what, exactly, women, liberals, and the intellectually humble are doing that makes their arguments more persuasive.

## Materials and methods

The persuader sample was a convenience sample of Democrats and Republicans on Prolific (https://app.prolific.co/), and the judge sample was a nonprobability sample quota matched to US census demographics (age, education, ethnicity, and region) and political identification from Forthright Access (https://forthrightaccess.com/). The three-item measure of persuasiveness (11) asked judges on 1–7 Likert items “how strong is their argument?” (“very weak” to “very strong”), “how valid is their argument?” (“not at all valid” to “extremely valid”), and “how satisfied are you with their argument?” (“not at all” to “extremely”). Intellectual humility was measured with six items (23), economic and social political ideology with 12 items (24), and political party identification with six items (25). See Supplementary material for complete methodological details.

## Supplementary Material

pgad143_Supplementary_Data

## Data Availability

All study materials, anonymized data, analysis files, and preregistrations are on the Open Science Framework (https://osf.io/453cp). The analyses herein were preregistered as exploratory (see https://osf.io/rbgz4).

## Footnotes

1 Judges also rated the arguments on other qualities unrelated to persuasiveness, which are outside the scope of this manuscript. See Supplementary material for details.
