# Correction to: Women, the intellectually humble, and liberals write more persuasive political arguments

**DOI:** 10.1093/pnasnexus/pgad272

**Published:** 2023-08-22

This is a correction to: Jeffrey Lees and others, Women, the intellectually humble, and liberals write more persuasive political arguments, PNAS Nexus, Volume 2, Issue 5, May 2023, pgad143, https://doi.org/10.1093/pnasnexus/pgad143

The Funding section of this manuscript has been updated to include the following statement:

‘This publication was supported by the Princeton University Library Open Access Fund.’

