# Supplementary material for: Women, the intellectually humble, and liberals write more persuasive political arguments
Source: PNAS Nexus. 2023 Apr 25;2(5):pgad143. doi: 10.1093/pnasnexus/pgad143 (PMC10184441; doi:10.1093/pnasnexus/pgad143)
Supplement: pgad143_Supplementary_Data [file pgad143_supplementary_data.docx]

#### **Supplemental Materials for Lees, Todd, & Barranti (2023)**

##### **Detailed Methods**

##### ***Open Science and Preregistrations***

All study materials, anonymized data, and analysis files can be found on the Open Science Framework (<https://osf.io/453cp>). All aspects of data collection (such as sample sizes) and analysis were preregistered, including for the persuader sample (<https://osf.io/6svnk>), the judge sample (<https://osf.io/rbgz4>). The analysis of what variables predict persuasiveness was preregistered (see “Exploratory Analyses” in <https://osf.io/rbgz4>), however no hypotheses regarding what variables would predict persuasiveness were given. Lastly, a portion of the present study is related to the measurement and analysis of meta-perception accuracy, i.e. can persuaders accurately predict how others will judge their argument. The analysis of meta-perception accuracy is presented in a separate manuscript. Here, the focus is solely on predicting persuasiveness.

##### ***Participants***

*Persuader Sample*: A preregistered convenience sample of 605 Democrats and Republicans, evenly split based on a prescreen, were recruited on the Prolific survey platform (Palan & Schitter, 2018) in July 2021. Two participants wrote nothing, two participants failed the comprehension check, and two participants took the survey twice and repeated their argument verbatim (i.e., four responses total), leaving 597 usable responses. Then, in a preregistered procedure (<https://osf.io/6svnk>), all 597 arguments were independently read by three members of the research team and rated on a binary scale for coherence (the argument can be read and lacks grammatical errors that compromise the ability to understand what is written), and normativity (the argument has a minimal normative component and meets the basic definition of an argument). Only if all three researchers independently rated an argument as lacking one or both of these qualities was it removed, and this procedure led to the removal of three arguments, leaving a final *N*_persuaders_ = 594, 298 Democrats/296 Republicans, 330 Male/260 Female/4 Non-binary or unlisted, *M*_age_ = 36.5, *SD*_age_ = 12.5.

*Judge Sample*: We sought to collect a preregistered sample of 3,300 judges from a non-probability US representative sample, quota matched to census-based demographics (age, education, ethnicity, region) and political identification, through a Forthright Access survey panel (<https://forthrightaccess.com/>) in April-May 2022. Participants had to pass a basic comprehension check at the beginning of the survey to continue, and 525 participants failed this check while 3304 passed and completed the survey. Then, in a preregistered procedure (<https://osf.io/rbgz4>), participants were removed from all analyses is they *both* failed at attention check in the middle of the survey and were in the bottom quartile of survey duration, as these two factors together indicate an undesirable level of participant inattentiveness. 173 participants (5.2% of the sample) met these criteria and were removed from all analyses, leaving a final sample of *N*_judges_ = 3,131, 1574 Democrats/1293 Republicans/263 True Independents, 1548 Male/1552 Female/31 Non-binary or unlisted, *M*_age_ = 47.9, *SD*_age_ = 15.3.

##### ***Procedures***

*Persuader Procedure*: After providing informed consent and indicating political party identification, participants received the prompt to write a persuasive political argument, which asked them to write at least four sentences on any political topic they wished. A between-subjects manipulation of the target of persuasion was embedded within the prompt. Participants were randomly assigned to try and persuade an in-party member, out-party member, or the “average American.” Other than shifting target labels, the prompt was identical across conditions. In the prompt participants were informed that their argument would be read and rated by a people in the group they are assigned to persuade, and that these judges will be informed of their (the persuader’s) party-leaning. Participants are also informed of the incentive: that their pay will double if their argument is in the top 25% of persuasive arguments, based on the judges’ ratings at a future date. After writing their argument participants responses to all measures (see below) at the self-level (their perceptions of their own arguments, and self-reports of their own opinions) and meta-level (how they think the “average” member of their target groups would perceive them and their argument). Whether participants received the self or meta items first was randomized, and within those blocks the order of the measures was also randomized. The exact text of their argument was visible to participants at the top of each page as they completed the self- and meta-items. Lastly, participants responded to demographic questions, provided open comment, and the survey ended. The survey was advertised as taking 15 minutes (*Median*_duration_ = 16 minutes 34 seconds), and participants were compensated $3.25 (+ $3.25 if in top 25% of persuasiveness).

*Judge Procedure:* After providing informed consent and indicating political party identification, participants received instructions that they would be reading six written political arguments (described “sincere attempts at political persuasion”) from individuals in a past survey. Judges were informed that persuaders had discretion over the topic they wrote on. Judges were then randomly assigned to six of the 594 arguments: four arguments written for their party (e.g. a Republican judge saw four arguments randomly selected from all Democratic persuaders assigned to persuade Republicans and all Republican persuaders assigned to persuade Republicans), and two arguments written for the “average American.” Judges who were true independents instead read just four arguments written for the “average American.” Six arguments were chosen based on survey length limitations and to ensure that each of the 594 arguments were judged by at least 30 judges. For each argument the judges rated it on the same measures (detailed below) for which persuaders provided self- and meta-ratings. Lastly, provided open comment and the survey ended. The survey was advertised as taking 20 minutes (*Median*_duration_ = 28 minutes 9 seconds), and participants were compensated $2.50 + 1 Loyalty Credit (worth $0.67 in Forthright Access’ internal system).

*Measures*: Persuaders were asked at the self-perception and meta-perception level, and judges at the perception level, all the following about the arguments and the persuaders themselves, all using the exact same 7-point Likert scales and near-identical question language across the levels (self- and meta-items shifted the referent, i.e., “you” versus “they”, while the language for meta-items and judges perceptions were identical). The measures were perceived effort (3-item, e.g., “How much energy did they put into generating their arguments”) and persuasiveness (3-item, e.g. “How strong is their argument”) (both from Briñol et al., 2012), five measures developed by the researchers, clarity (3-item, e.g., “The argument is clear”), reasonableness (3-item, e.g. “The argument is sensible”), typicality (3-item, e.g. “The argument is made by many people”), attitude change (3-item, e.g. “The argument will lead others to reconsider their opinions”), and conflict caused (3-item, e.g. “The argument will make people angry”), intellectual humility (6-item, e.g. “I reconsider my opinions when presented with new evidence”) (from Leary et al., 2017), economic and social political ideology (12-item, e.g. “How positive or negative do you feel about limited government”) (from Everett, 2013), and identification with the Democratic and Republican parties (6-item, e.g. “I feel connected to Democrats”) (from Lau et al., 2016). The full scales used in both samples, in simple, easy to read formats, can be found on the OSF: Persuader measures (https://osf.io/2aqnd), Judge measures (https://osf.io/e7ndm)

##### **Further Analysis Details**

***Linear Mixed Effects Models***

Models were run using *lme4* (Bates et al., 2015), and *P*-values and degrees of freedom approximation was done using Welch–Satterthwaite approximation via the *lmerTest* package (Kuznetsova et al., 2017). All linear variables were grand mean-centered and *z*-scored, as no predictors were crossed with both judge and persuader, which would have required cluster-centering (Enders & Tofighi, 2007). See online analysis code for full model specifications.

***Education Effect***

In the baseline model, education is associated with persuasion, *F*(6, 572.6) = 2.79, *P* = 0.011. An investigation of the mean perceived persuasion across the educational categories suggested, and a test of orthogonal polynomial contrasts supports, β = -2.99 [-5.49, -0.48], *t*(606) = -2.34, *P* = 0.020, that education had a negative quadratic relationship with persuasion, such that those with middling education (“Some college,” and those with 2 and 4 year degrees) were the most persuasive, while those with graduate degrees and no college degrees were less persuasive. Indeed, marginal mean estimates from the baseline model found the education group with the highest persuasiveness were those with 2-year college degrees (*M* = 4.59, 95% CI = [4.22, 4.97]), while those with Masters (*M* = 4.20, 95% CI = [3.89, 4.52]) and Doctorates (*M* = 4.05, 95% CI = [3.64, 4.46]) were lower than (but statistically indistinguishable from) those with high school degrees only (*M* = 4.26, 95% CI = [3.91, 4.60]).

***Sentiment Analyses***

Sentiment analyses were conducted using *TextAnalyzer* (Berger et al., 2020), which was supplied the raw text of each argument. Grade level was calculated using Flesch-Kincaid Grade Level (Kincaid et al., 1975), emotional valence was based on Rocklage et al. (2018), fear was based on Mohammad & Kiritchenko (2015), and dominance based on Mohammad (2018). Grade level was positively associated with persuasion, *r*(592)*_persuasion_* = 0.20, *P* < 0.001, liberalism, *r*(592)*_liberal_* = 0.10, *P* = 0.012, and identifying as female, *d_female_* = 0.27, *t*(405.92) = 3.17, *P* = 0.002. Emotional valence was positively associated with persuasion, *r*(592)*_persuasion_* = 0.11, *P* = 0.007, and liberalism, *r*(592)*_liberal_* = 0.09, *P* = 0.032. Fear was positively associated with persuasion, *r*(592)*_persuasion_* = 0.09, *P* = 0.033, and liberalism, *r*(592)*_liberal_* = 0.08, *P* = 0.047. Dominance was negatively associated with persuasion, *r*(592)*_persuasion_* = -0.13, *P* = 0.002, and identifying as female, *d_female_* = -0.30, *t*(586.10) = -3.72, *P* < 0.001.

These sentiments were chosen, amongst the many available from *TextAnalyzer*, based on exploratory analyses of which sentiments showed gender differences and/or correlations with ideology at base. See online analysis materials on the OSF. Sentiment analyses were not preregistered.

Table S1 presents the gender by topic cross-section. To examine whether a particular topic was perceived as more persuasive on average, we regressed the mean perceived persuasion by argument onto a categorical fixed effect for topic, using ordinary least square regression. We then computed marginal means contrasts for each topic relative to the average perceived persuasiveness of all other topics. The topics of Healthcare, *b* = 0.32, *t*(750) = 2.69, *P* = 0.007, and Economic Inequality, *b* = 0.28, *t*(750) = 2.49, *P* = 0.013, were more persuasive than average, and the “Other” categories was less persuasive than average, *b* = -0.21, *t*(750) = -2.12, *P* = 0.034. These all became statistically insignificant when *P*-values were corrected for multiple comparisons, so we caution against confidence in these findings.

When dummy variables were added to the baseline linear mixture model indicating the presence of the Healthcare, β = 0.08 [-0.02, 0.18], *t*(565.32) = 1.56, *P* = 0.120, Economic Inequality, β = 0.06 [-0.03, 0.15], *t*(555.07) = 1.24, *P* = 0.214, and “Other” categories, β = -0.06 [-0.13, 0.03], *t*(566.31) = -1.16, *P* = 0.245, none were statistically significant and the gender effect persisted, β = 0.08 [0.01, 0.14], *t*(562.66) = 2.36, *P* = 0.019.

***Table S1***

| **Topic** | **Male** | **Female** | **Non-binary/unlisted** |
| --- | --- | --- | --- |
| abortion | 22 | 41 | 0 |
| climate_change | 15 | 16 | 0 |
| covid | 17 | 16 | 0 |
| crime | 12 | 17 | 0 |
| economic_inequality | 31 | 44 | 2 |
| economy | 104 | 44 | 1 |
| ethnic_inequality | 20 | 8 | 0 |
| foreign_policy | 21 | 14 | 0 |
| gun_policy | 32 | 20 | 0 |
| health_care | 31 | 37 | 0 |
| immigration | 23 | 30 | 0 |
| other | 76 | 38 | 0 |
| race | 20 | 8 | 0 |
| supreme_court | 0 | 3 | 1 |

Table S1: Crosstab of researcher-coded topics and gender of persuader.

***Category Coding***

The coding scheme for the categories was chosen by the research team after collecting all the arguments and was not preregistered. It was taken from a 2020 Pew Survey (Pew Research Center, 2020) about the topics most important to US voters in the 2020 US Presidential Election. The coding of each argument was done by Author #2, based on their discretion of the topic(s) within each argument.

***Structured Topic Modeling***

Topic modeling was performed using the *stm* package in R (Roberts, Stewart, & Tingley, 2019). The corpus of arguments was first processed by making all words lowercase, removing punctuation, numbers, and stop words, and stemming all words. Diagnostic values were then derived for all models of topic three through fifteen. See Figure S1 for diagnostic values. Ten was chosen as the most appropriate number of topics.


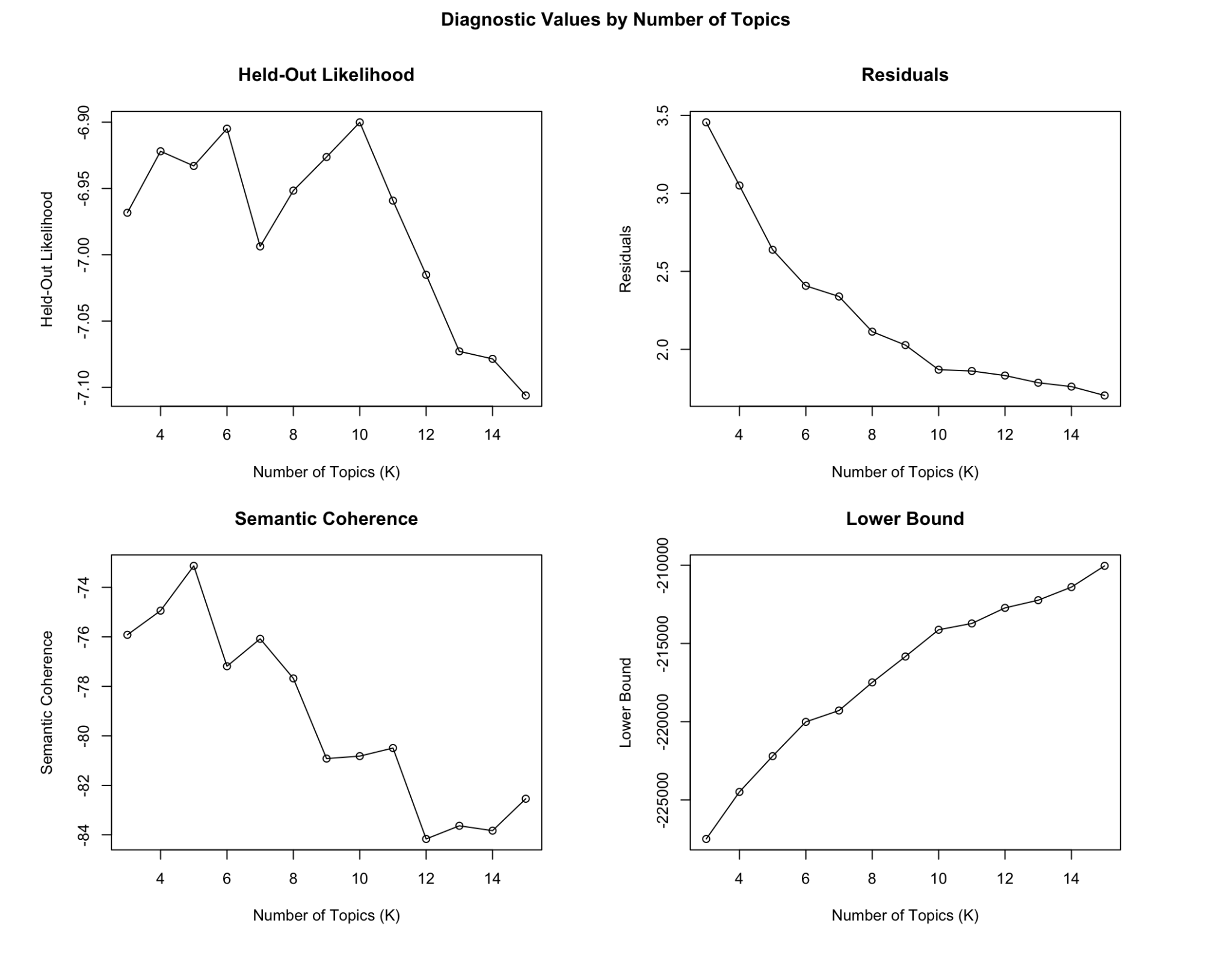


Figure S1: Diagnostic values for topic models, modeled with 3-15 topics, performed on corpus of argument.

##### **Supplemental References**

Bates, D., Maechler, M., Bolker, B., & Walker, S. (2015). Fitting linear mixed-effects models using lme4. Journal of Statistical Software, 67(1), 1–48. <https://doi.org/10.18637/jss.v067.i01>

Berger, J., Sherman, G., & Ungar, L. (2020). TextAnalyzer.

Briñol, P., McCaslin, M. J., & Petty, R. E. (2012). Self-generated persuasion: Effects of the target and direction of arguments. Journal of Personality and Social Psychology, 102(5), 925–940. <https://doi.org/10.1037/a0027231>

Enders, C. K., & Tofighi, D. (2007). Centering predictor variables in cross-sectional multilevel models: A new look at an old issue. Psychological Methods, 12(2), 121–138. <https://doi.org/10.1037/1082-989X.12.2.121>

Everett, J. A. C. (2013). The 12 item social and economic conservatism scale (SECS). PLoS ONE, 8(12), e82131. <https://doi.org/10.1371/journal.pone.0082131>

Kincaid, J. P., Fishburne Jr, R. P., Rogers, R. L., & Chissom, B. S. (1975). Derivation of new readability formulas (automated readability index, fog count and flesch reading ease formula) for navy enlisted personnel. Naval Technical Training Command Millington TN Research Branch.

Kuznetsova, A., Brockhoff, P. B., & Christensen, R. H. B. (2017). lmerTest package: Tests in linear mixed effects models. Journal of Statistical Software, 82(13), 1–26. https://doi.org/10.18637/jss.v082.i13

Lau, T., Morewedge, C. K., & Cikara, M. (2016). Overcorrection for social-categorization information moderates impact bias in affective forecasting. Psychological Science, 27(10), 1340–1351. <https://doi.org/10.1177/0956797616660292>

Leary, M. R., Diebels, K. J., Davisson, E. K., Jongman-Sereno, K. P., Isherwood, J. C., Raimi, K. T., Deffler, S. A., & Hoyle, R. H. (2017). Cognitive and interpersonal features of intellectual humility. Personality and Social Psychology Bulletin, 43(6), 793–813. <https://doi.org/10.1177/0146167217697695>

Mohammad, S. M. (2018). Obtaining reliable human ratings of valence, arousal, and dominance for 20,000 English words. Proceedings of the 56th Annual Meeting of the Association for Computational Linguistics (Volume 1: Long Papers), 174–184. <https://doi.org/10.18653/v1/P18-1017>

Mohammad, S. M., & Kiritchenko, S. (2015). Using hashtags to capture fine emotion categories from tweets. Computational Intelligence, 31(2), 301–326. <https://doi.org/10.1111/coin.12024>

Palan, S., & Schitter, C. (2018). Prolific.ac—A subject pool for online experiments. Journal of Behavioral and Experimental Finance, 17, 22–27. <https://doi.org/10.1016/j.jbef.2017.12.004>

Pew Research Center. (2020). Election 2020: Voters are highly engaged, but nearly half expect to have difficulties voting. <https://www.pewresearch.org/politics/wp-content/uploads/sites/4/2020/08/PP_2020.08.13_Voter-Attitudes_FINAL1.pdf>

Roberts, M. E., Stewart, B. M., & Tingley, D. (2019). stm: An R Package for Structural Topic Models. Journal of Statistical Software, 91(2). https://doi.org/10.18637/jss.v091.i02

Rocklage, M. D., Rucker, D. D., & Nordgren, L. F. (2018). The Evaluative Lexicon 2.0: The measurement of emotionality, extremity, and valence in language. Behavior Research Methods, 50(4), 1327–1344. <https://doi.org/10.3758/s13428-017-0975-6>
